# Targeting Unconventional Host Components for Vaccination-Induced Protection Against TB

**DOI:** 10.3389/fimmu.2020.01452

**Published:** 2020-07-24

**Authors:** Elisa Nemes, Shabaana A. Khader, Rosemary V. Swanson, Willem A. Hanekom

**Affiliations:** ^1^South African Tuberculosis Vaccine Initiative, Division of Immunology, Department of Pathology and Institute of Infectious Disease and Molecular Medicine, University of Cape Town, Cape Town, South Africa; ^2^Department of Molecular Microbiology, Washington University School of Medicine, St. Louis, MO, United States; ^3^Africa Health Research Institute, Durban, South Africa

**Keywords:** tuberculosis, NK cells, trained immunity, B cells, Th17 Cells

## Abstract

The current tuberculosis (TB) vaccine, Bacille Calmette-Guerin (BCG), is effective in preventing TB in young children but was developed without a basic understanding of human immunology. Most modern TB vaccine candidates have targeted CD4^+^ T cell responses, thought to be important for protection against TB disease, but not known to be sufficient or critical for protection. Advances in knowledge of host responses to TB afford opportunities for developing TB vaccines that target immune components not conventionally considered. Here, we describe the potential of targeting NK cells, innate immune training, B cells and antibodies, and Th17 cells in novel TB vaccine development. We also discuss attempts to target vaccine immunity specifically to the lung, the primary disease site in humans.

## Introduction

Only one vaccine is currently licensed to prevent tuberculosis (TB), i.e., Bacillus Calmette-Guerin (BCG). This vaccine is safe and effective in protecting young children primarily against disseminated forms of the disease and, to a lesser extent, against pulmonary TB (1). BCG's protection against TB disease at older ages has been variable and mostly poor (1); however, it was recently shown that BCG may have protective efficacy against *Mycobacterium tuberculosis* (Mtb) infection, as defined by sustained interferon-γ release assay (IGRA) conversion, in adolescents who had been IGRA negative at the time of vaccination (2). BCG was developed nearly a century ago, with very limited understanding of human immunology. The experience with BCG demonstrates that empiric vaccine discovery and development may prove successful, sometimes without a grasp of underlying protective host determinants.

As our knowledge of human immunology has expanded, modern vaccine development has relied on induction of specific immune responses that have been shown or are thought to be essential for protection against a specific disease. Although there has been remarkable recent progress in our understanding of the host responses against TB (3), the critical determinants for vaccine-induced protection against infection and disease in humans remain unknown.

The modern, post-BCG era of TB vaccine discovery and development is about 20 years old. As recently reviewed (3), virtually all subunit or viral vectored candidate TB vaccines tested clinically rest on a hypothesis that CD4^+^ T cell (and in some cases CD8^+^ T cell) induction of interferon-gamma (IFN-γ) is a host determinant of protection. Therefore, most Mtb antigens included in these novel vaccines were selected based on their capacity to induce IFN-γ in peripheral blood mononuclear cell cultures from persons latently infected with Mtb and then evaluated in preclinical models. This approach may have been suboptimal, for two reasons. First, not all antigens recognized in latently infected persons may be associated with protective immune responses, although most people never progress to active TB disease. Second, so-called immunodominant antigens identified in these screens have been shown to demonstrate the least variation among global Mtb strains, compared with other Mtb antigens, suggesting a lack of evolutionary immune pressure and that a host response to these antigens may even hold advantage for the pathogen (4). Regardless, the recent demonstration that a candidate subunit vaccine, M72/AS01E, which contains two immunodominant antigens that induce IFN-γ, may protect latently infected young adults against progression to disease (5) is evidence that this approach may hold promise. Of course, it is not clear whether M72/AS01E's potential protection involves CD4^+^ T cell IFN-γ production or whether an alternate mechanism is at play.

In contrast to subunit or viral vectored vaccines, it is hypothesized that whole-cell TB vaccine candidates would include the full complement of antigens that are important for protection, through both known and unidentified immune mechanisms. The approaches have included modifying BCG to potentially enhance immunity induced by the BCG—to create VPM1002 (6), e.g.,—and attenuating Mtb—to generate MTBVAC (7), e.g., The approach, therefore, returns to some degree of empiricism by hypothesizing that the primary pathogen is likely to induce more immune components critical for protection.

Both the adolescent BCG and adult M72/AS01E trials mentioned involved prospective collection and storage of blood, which would, for the first time, allow studies to identify human correlates and mechanisms of vaccination-induced protection. These ongoing efforts, led by the Bill & Melinda Gates Foundation Medical Research Institute (N. Frahm; personal communication), will be informed by breakthroughs from non-human primate studies, in which TB most closely resembles human disease, where intravenous administration of BCG has been shown to result in sterilizing protection against Mtb challenge (8), for example. The vaccine dose can now be downtitrated to allow breakthrough disease for subsequent elucidation of correlates or mechanisms of protection by comparing protected and unprotected animals. Similarly, excellent protection induced by a CMV-based vaccine in non-human primates will allow a similar analysis (9).

**Figure 1:**
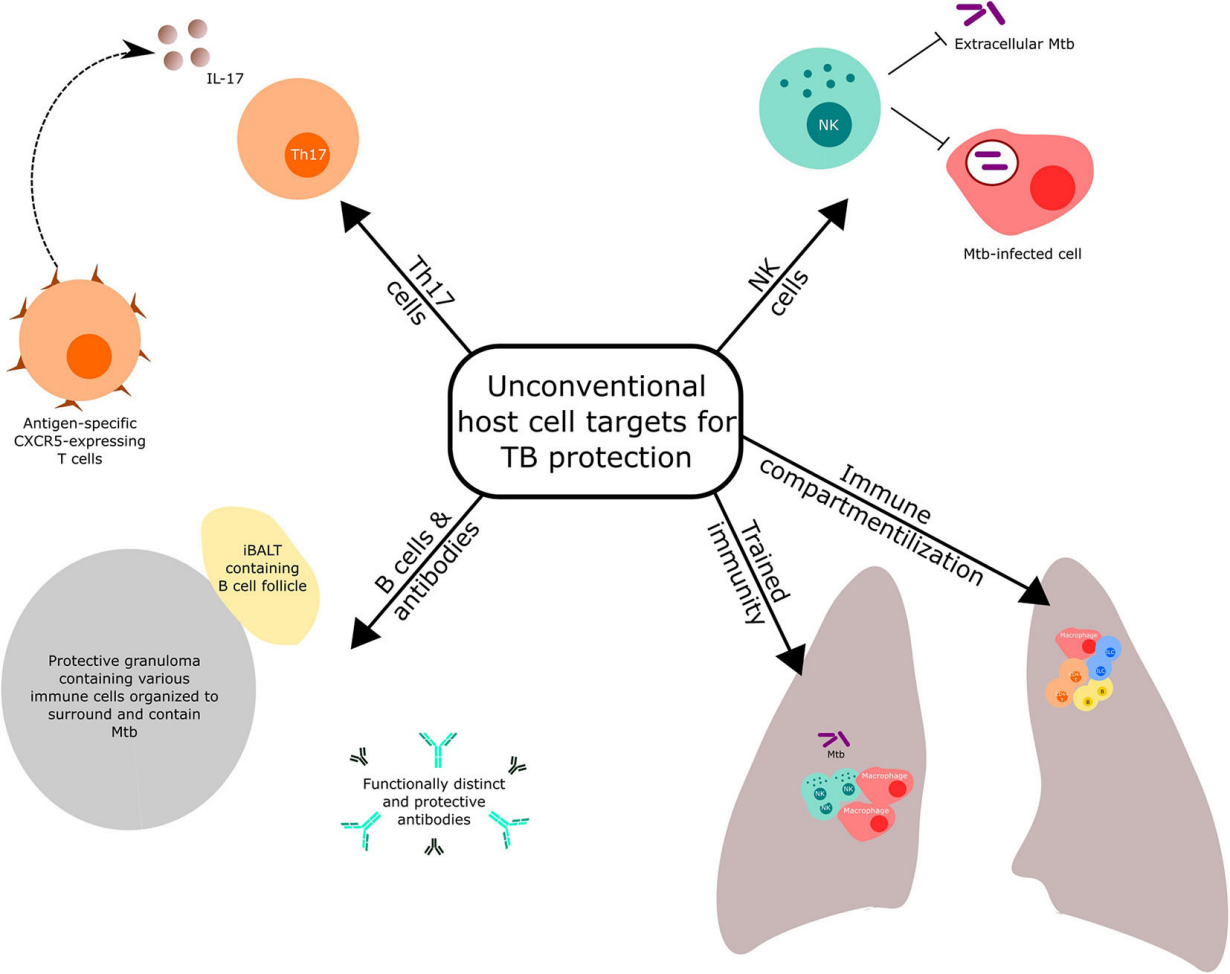
Unconventional host cell targets for protection against tuberculosis.

Reviews covering conventional Th1 and innate responses targeted by novel vaccination strategies against TB have been recently published (3, 10). Here, we highlight selected host immune components (Figure 1), other than the conventional T cell response mentioned above, that have received recent prominence for potential targeting in TB vaccine discovery and development. We also discuss the importance of optimal immune compartmentalization of vaccination-induced responses.

## NK Cells

NK cells are involved in immune responses to a variety of pathogens. While deficiency of NK cells alone is not associated with higher risk of mycobacterial diseases in humans (11), evidence suggests that NK cells actively participate to responses against Mtb in cooperation with other arms of the immune system.

Purified NK cells can directly interact with extracellular mycobacteria by binding cell wall components through TLR-2 and NKp44 (12) and become functionally activated in the presence of an appropriate cytokine milieu (13). More commonly, NKs recognize autologous cells infected by viruses or intracellular pathogens and respond to cytokines produced by myeloid cells and antigen-specific T cells, while their cytotoxic activity can be directed by pathogen-specific antibodies (14). IL-12 is a potent inducer of IFN-γ secretion by NK and other cells, and genetic mutations of IL-12Rα are associated with mendelian susceptibility of mycobacterial disease (15). NK cells can also be activated by IL-18 produced by Mtb-infected myeloid cells and contribute to early protection against Mtb in a mouse model (16). The main NK effector functions that have been implicated in antimycobacterial immunity include lysis of Mtb-infected alveolar macrophages, cytokine production, direct bacterial killing, production of antimicrobial mediators, and immune regulation [recently reviewed (17)]. In mice, NK cells seem dispensable in an immunocompetent host (18) but represent the main source of IFN-γ in T cell–deficient animals, contributing to the containment of Mtb early after infection (19).

In humans, NK cell abundance and functional profiles change during different phases of Mtb infection and progression to TB disease (20). In peripheral blood, NKs are more abundant in healthy Mtb-infected individuals compared to uninfected donors, decrease during progression to active TB disease, and are restored upon successful treatment. In patients with active TB disease, frequencies of NK in blood are inversely correlated with pulmonary inflammation measured by PET-CT (20), while NKs are recruited to lung lesions (13). NK cells expressing IFN-γ and IL-22 in response to cytokines and mycobacterial antigens were found in the pleural fluid of TB patients (21), and IL-22 production by NK cells has been associated with inhibition of Mtb growth in macrophages in healthy individuals (22). The various studies reporting impairment of NK effector functions, particularly cytotoxicity, in TB patients were recently reviewed (17, 23): taken together, NK cells migrate to the site of active Mtb replication, where they contribute to host responses against TB, although their specific role remains to be established.

Despite being traditionally considered innate cells, a growing body of evidence suggests that NK cells exhibit “memory-like” features, characterized by more rapid and robust responses upon secondary exposure to the same pathogen (23). In a mouse model, memory-like NK cells were induced by BCG vaccination, and adoptive transfer of CD27^+^ NK cells isolated 6 months post-vaccination was associated with lower Mtb burden upon challenge of recipient mice; on the other hand, NK cells isolated from unvaccinated donors were not associated with protection (24). In humans, neonatal BCG vaccination of naïve infants induced BCG-reactive IFN-γ-producing NK responses, as did BCG revaccination of Mtb-infected adults, in whom responses were sustained for at least 1 year post-vaccination (25). Such NK responses were correlated with the frequencies of BCG-specific, IL-2-producing CD4^+^ T cells, but were completely abrogated by blocking IL-12 and IL-18. Indeed, another study showed that BCG vaccination was associated with increased pro-inflammatory NK responses to unrelated pathogens, suggesting that BCG could “train” NK cells independently from bystander activation from antigen-specific CD4^+^ T cells (26). Whether BCG “training” of NKs is mediated via epigenetic modifications of NK cells themselves or via enhanced IL-12 and IL-18 production by myeloid cells remains to be determined. Human vaccination with M72/AS01_E_ was also associated with increased frequencies of IFN-γ-producing NK cells in responses to M72 peptide stimulation of PBMC, and was positively correlated with frequencies of M72-specific, IL-2-producing CD4^+^ T cells (27). In addition to direct Mtb binding and bystander activation via antigen-specific CD4 T cells, NK effector functions could also be directed specifically to Mtb through binding of Mtb-specific antibodies. Indeed, antibodies purified from healthy Mtb-infected individuals showed enhanced capacity to induce NK cell activation and ADCC compared to patients with active TB disease (28). Additionally, while other innate lymphoid cells such as Group 3 ILCs have also been recently implicated in protective immunity to TB (29), their role in vaccine-induced immunity is unknown and needs to be fully explored.

Taken together, these studies suggest that NK cells actively contribute to immune responses against Mtb, can be modulated by vaccination, and can be specifically directed to respond to Mtb. While the mechanisms underlying these observations remain to be established, induction of NK responses by vaccination and their role in supporting and amplifying adaptive immunity during the early stages of Mtb infection and progression to TB disease deserve to be considered in immune correlates of protection studies built on recent efficacy trials of BCG (2) and M72 vaccines (5).

## Trained Immunity

Recent studies have highlighted further roles for innate immune cells in protection against reinfection. The term “trained immunity” refers to innate immune cells undergoing reprogramming that ultimately leads to an increase in antimicrobial function such as phagocytosis, production of proinflammatory cytokines, or killing (30, 31). Thus, cells of the innate immune system that have been trained by prior immunologic stimuli would be able to generate a more potent response and clear pathogens more efficiently upon secondary encounter with non-related pathogens. Khader et al. (10) review the complex interplay of immunological signals, which ultimately leads to the functional reprogramming of these innate immune cells.

Many cells that have been implicated in trained immunity, including monocytes and macrophages, are relatively short-lived, so this cannot fully explain how trained immunity appears to be relatively long-lived. One possible explanation is that hematopoietic stem cells (HSCs) are also trained in the process. Expanded HSC populations were found in models of acute and chronic bacterial infections (32, 33) and were associated with trained immunity (34, 35), although the mechanism through which HSCs are activated and the duration of trained immunity remain to be fully elucidated. In a mouse model of BCG vaccination, the protective effects of trained immunity on HSCs were through a type II IFN-dependent mechanism (34).

As the innate immune system is considered among the first checkpoints through which a pathogen must pass, trained immunity presents a new avenue to explore in vaccine design. BCG may provide an example on how innate training could impact vaccination. It has recently been reported that BCG-vaccinated household contacts of patients with active TB disease were less likely to convert their IGRA to positive, compared with household contacts who had not been BCG vaccinated, suggesting an early clearance of Mtb (36). Additionally, BCG vaccination in West Africa was associated with increased childhood survival attributable not solely to its effects against TB (37). BCG-associated, non-specific protection against other pathogens such as respiratory syncytial virus, yellow fever, and malaria has also been reported (38–43). It is interesting to note that BCG is also used in the treatment of bladder cancer (44, 45), an effect far removed from protection against TB. Many of these effects have been associated with increased production of pro-inflammatory cytokines (2, 36, 46–50). It should be noted, however, that several studies have published results refuting the non-specific benefits of BCG, notably Haahr et al. (51), who found no evidence that neonatal BCG vaccination reduced childhood morbidity to other infectious diseases in a population in Greenland, and Stensballe et al. (52) who found that BCG vaccination did not reduce the risk for somatic acquired disease in children in Denmark.

Trained immunity has untapped potential for improving vaccines, allowing both innate and adaptive systems to be engaged for early clearance and long-term protection. Regardless, our current knowledge of how the innate system is trained, durability of trained effects, and how we can train immune cells within the context of vaccines remains suboptimal. The specific application of this field to TB vaccine development has recently been reviewed (10).

## B Cells and Antibodies

Virtually all vaccines that have been introduced successfully into public health mediate protective effects by inducing antibodies, which classically act through neutralization. Whether B cells and/or antibodies contribute to protection against TB, and to vaccination-induced protection mediated by BCG, remains unclear. The humoral response to BCG vaccination was recently reviewed (53); there is suggestive but inconclusive evidence for a role in protection against TB disease; recently, the M72/AS01E vaccine candidate has been shown to be a potent inducer of antibodies (5). A systematic investigation into B cell and antibody contribution to BCG-induced protection has not been undertaken with modern immunological tools, although the above-mentioned effort to identify the correlates of BCG-induced prevention of Mtb infection in adolescents may shed some light.

It is abundantly clear that B cells and antibodies are induced in the course of Mtb infection and disease. Antigen-specific B cells that can proliferate are present in granulomas induced by infection and disease in humans (54). In TB granuloma B cell follicles, CXCR5-expressing CD4^+^ T cells colocalization near Mtb-infected macrophages results in better control of the pathogen in mice (54, 55). Protection induced by a novel TB vaccine, MtbΔsigH, against Mtb challenge in the macaque was associated with accumulation of highly organized bronchus-associated lymphoid tissue (iBALT), consisting of CD20^+^ B cells (and CCR5+ cells T cells) in granulomas (55). Other ongoing non-human primate studies that aim to characterize granulomas that control, vs. permit, Mtb bacilli should enhance our understanding of whether these cells contribute to the control of the pathogen (56).

A recent review summarized evidence that antibodies play a role in protection against TB (57). Four emerging themes were described. First, antibodies from latently infected persons were distinct from and functionally more protective than those from patients with TB disease (28). Second, the antibodies also display unique glycosylation patterns, which are associated with distinct immune cell function, i.e., in latently infected persons, IgG glycosylation profiles included lesser “inflammatory” (e.g., lesser agalactosylated) and more “anti-inflammatory” (e.g., higher di-galactosylated and higher sialic acid) patterns than those isolated from TB patients (28). As mentioned above, latency may not always reflect host control of Mtb infection; whether antibody functional and phenotypic features are critical effector mechanisms of mycobacterial control must still be shown. The third theme was that antibody isotype appears to be important in protection, with IgA apparently most protective, particularly at the mucosal surface (57), while the fourth theme was that antibodies generated during Mtb infection and disease target not only cell wall components of the pathogen but also non-surface antigens (57).

Finally, a recent clinical study compared antibody responses between persons who had been highly exposed to Mtb but remained tuberculin skin test or IGRA negative, suggesting “resistance” to infection, and those who converted these tests to positive. The former group was shown to possess IgM and class-switched IgG antibody responses, indicating that exposure did indeed occur (58, 59). It is tempting to speculate that this antibody response could have contributed to “protection” against infection, but this could be challenged by the observation that the T cell response to Mtb antigens detected in the “resistant” individuals did involve IFN-γ-independent T cell responses, therefore indicating that they could well have been infected.

No TB vaccine candidates have been developed to date to specifically induce NK cells and innate immune training, B cells, or antibodies. Since these immune responses are partially induced by BCG and/or M72/AS01E, ongoing studies may shed some light on the contribution of such responses to protection against TB.

## Th17 Cells

IL-17A is the best characterized among the IL-17 cytokine family members and signals through the heteromeric receptor IL-17R, comprising of IL-17RA and IL-17RC. Upon exposure to Mtb, innate myeloid cells induce cytokines such as IL-23 and IL-1β, which initiate the differentiation and polarization of naïve CD4 T cells toward T helper cell type 17 (Th17) cells (60). Th17 cells are the primary producers of IL-17 during TB (60), but can also co-produce IL-22, IL-21, tumor necrosis factor-α (TNF-α), and granulocyte macrophage colony stimulating factor (GM-CSF) (61, 62). IL-17 can also be produced by γδ T cells (63) and group 3 innate lymphoid cells (ILC3s) (29) to mediate early innate immune responses following Mtb infection, while invariant natural killer T (iNKT) (64) cells, innate Th17 cells (iTh17) (65), and NK (66) cells can also produce IL-17 following stimulation of TGF-β, IL-1β, IL-6, IL-23, or α-galactoceramide (α-Galcer) (67). In the context of vaccination and Mtb challenge, IL-23 and IL-17 gene deficient mice are not protected upon vaccination with BCG (68) nor are subunit vaccines delivered parenterally (69) or mucosally (70, 71), suggesting an important role for the IL-23/IL-17 axis in driving vaccine-induced protective immunity against Mtb infection. This is in contrast to studies using IL-12 or IFN-γ-/- mice that are still protected when vaccinated with either BCG or subunit vaccine candidates and challenged with Mtb (68, 69, 71).

IL-17 produced by Th17 cells can induce T cell attracting chemokines including CXCL-9-11 for rapid recruitment of protective antigen-specific T cells to the lung (69). IL-17 produced by Th17 cells (71) and ILC3s (29) can induce the expression of CXCL-13 to localize CXCR5 positive cytokine-producing T cells within lymphoid follicle-containing lung granulomas of Mtb infected mice. Furthermore, mucosal BCG vaccination of macaques that conferred sterilizing immunity upon Mtb challenge correlated with the presence of polyfunctional Th17 cells (72). These studies suggest that targeting Th17 cells may enhance vaccine-induced immunity for TB.

As Th17 cells and IL-17 in animal models correlate with protective TB vaccine responses, efforts have been made to identify adjuvants and delivery routes that can effectively induce Th17 responses (68–70, 72). Mucosal delivery of live BCG (70, 72) and subunit TB vaccines in enterotoxin-based adjuvants (73, 74) or TLR-based (75) or nano-emulsion adjuvants (76) preferentially induces Th17 responses in the lung and confers protection in animal models of TB. Despite a decade of data that Th17 cells have a protective role in vaccine-induced immunity against TB in mouse models, the functional role of IL-17 in the context of human TB and vaccination is still evolving. Studies suggest that IL-17 production during TB may be protective by inducing proinflammatory cytokines such as IL-12 and IFN-γ to limit pathogenesis within the host (77). Additionally, a single nucleotide polymorphism in the IL-17 promoter was recently linked with decreased IL-17 production and an increased association with TB (78, 79). Thus, future directions for targeting Th17 responses should involve careful analysis in ongoing studies to identify if Th17 cells, or IL-17 production in innate cells, are a correlate of protection. Simultaneously, development of safe Th17 adjuvants, preferably those that can be delivered mucosally with Mtb antigens to induce lung-resident Th17 and ILC3s, should also be pursued for TB vaccine design.

## Immune Compartmentalization

As the lung is the primary site of human disease, vaccination should induce appropriate immune responses in this organ for protection against TB. In mice, Mtb-specific CD4 T cells with the capacity to migrate from peripheral blood to the lung parenchyma are more protective against TB, compared with cells that recirculate in peripheral blood only (80). In non-human primates, CD153^+^ Mtb-specific CD4^+^ T cells are enriched in the airways, and their abundance in individual granulomas correlates inversely with the mycobacterial load (81). Even more granular features of CD4^+^ T cell responses may account for the heterogeneity across individual granulomas within a host (82). For example, only a minority of CD4^+^ T cells isolated from granulomas produce cytokines in responses to Mtb antigens, and only a handful appear positioned in proximity to Mtb-infected myeloid cells (83). Higher frequencies of Mtb-specific CD4^+^ T cells with balanced Th1/Th17 and IL-10 responses are associated with lower bacterial burden in individual granulomas (84). IL-22, an IL-17-related cytokine (85), has also been shown to be important for mycobacterial containment in the lung (86) and could play an underappreciated role in immunity against TB (87, 88). In both primates and humans, Mtb-specific CD4^+^ T cell responses measured in peripheral blood did not accurately reflect those detected in the infected lung (83, 84, 89). For example, in humans, lower frequencies of IL-22 expressing CD4^+^ T cells were found in the blood of TB patients, whereas BAL IL-22 protein levels were higher in TB cases, compared with healthy controls (87). Notably, innate lymphoid cells (ILCs) were also depleted in the blood of TB patients and were restored during treatment (29).

Since inducing appropriate immune responses in the lung seems important to protect against TB (90), increasing efforts have been made, and more are needed, to understand how vaccine administration could influence tissue localization of induced immunity. A recent study employing a repeated limiting dose Mtb challenge model in rhesus macaques showed that mucosal BCG vaccination induced protective immune responses against both Mtb infection and TB disease, including Th1/Th17 and IL-10 responses, which were only observed in the lung and not in blood (72). Further, as mentioned above, aerosol vaccination with attenuated Mtb showed superior protection compared to aerosol BCG against lethal challenge in macaques, which was associated with the induction of iBALT (55). Similarly, murine intranasal vaccination with BCG was associated with superior protection against TB when compared with subcutaneous BCG administration, and this protective effect was reduced by blocking IL-17 (70). Mucosal delivery of TB vaccines is being actively pursued, and clinical trials in humans showed that aerosol vaccination with the viral vectored MVA85A has the potential to induce more robust immune responses in the lung compared to intradermal administration (91, 92). Whether induction of more potent immune responses in the lung is sufficient for protection against TB remains to be established. For example, boosting intradermal BCG with an aerosol administration of an Ad5 vector containing various Mtb antigens did not enhance protection against TB in NHP (93). Similarly, mucosal boosting of parenteral vaccination with the subunit vaccine H56 did not enhance protection in mice, despite inducing significant increase in long-lived lung-resident T cells (94).

Identification of protective responses in animal models is critically important to inform hypotheses to be tested in TB vaccine trials in humans, but translation of such findings to clinical settings remains challenging. Collection of lung samples by bronchoalveolar lavage (BAL) could be critical to assess vaccine-induced immune responses at the site of Mtb entry. While it is not reasonable to obtain BAL samples on large numbers of participants enrolled in vaccine efficacy trials, collection of these samples from small immunogenicity cohorts is feasible (91, 92) and should be encouraged. Alternative and less invasive sampling methods, such as induced sputum, a routine practice for TB investigations (95), could be feasible even in large cohorts. Extensive profiling of the scarce immune cells present in sputum with single cell technologies enabling RNA and T cell receptor sequencing, as well as immunophenotyping by oligo-barcoded antibodies, could provide critical information about vaccine-induced immune responses in the human airways.

Although it seems clear that immune responses measured in peripheral blood poorly reflect lung immunity, it is currently unknown whether even immune responses detectable in BAL or sputum samples are representative of tissue resident immunity. Here, the non-human primate model could provide invaluable insight by correlating the detection of protective responses in granulomas, lymph nodes, and lung parenchyma to those measurable in BAL (96). Furthermore, even if the essential value of animal models resides in understanding mechanistic correlates of protection at the site of infection, measurement of peripheral blood biomarkers that correlate with protective immune responses in the lung should also be prioritized to enable translation to humans and facilitate clinical development of TB vaccine candidates.

## Conclusions

Recent clinical trials showing partial vaccine efficacy against established Mtb infection and progression to TB disease provide the first opportunity to discover immune correlates of protection in humans. It is important that such studies consider immune responses beyond conventional Th1, since mechanisms of protection against TB are likely complex and involve various components of the immune system, such as those reviewed here. Further, it is likely that profoundly different vaccines (i.e., live attenuated BCG and subunit M72/AS01E) trigger distinct immune responses, and that immune processes associated with the prevention of infection or disease would also be diverse.

## Author Contributions

EN, SK, RS, and WH wrote sections of the manuscript. All authors contributed to manuscript revision, read, and approved the submitted version.

## Conflict of Interest

The authors declare that the research was conducted in the absence of any commercial or financial relationships that could be construed as a potential conflict of interest.
